# Postoperative care in trachiasis surgery

**Published:** 2016

**Authors:** Esmael Ali

**Affiliations:** Research Fellow: International Centre for Eye Health, London School of Hygiene and Tropical Medicine, UK

**Figure:**
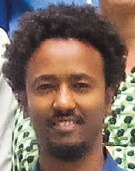
Esmael Ali

Postoperative care is an important aspect of trachomatous trichiasis (TT) surgical services. Follow-up visits should ideally take place on the first postoperative day (to remove the eye patch), after 8–14 days (to remove sutures; optional if absorbable sutures are used), at 3 months (to re-examine the operated lid for intermediate surgical outcomes), and then at or after six months.

An eye patch must by applied after surgery and should stay on overnight to prevent unconscious disruption of the wound by the patient while asleep. When removed the following day, the wound should be cleaned using gauze and normal saline and the operated eyelid should be examined. If the patient is not able to attend the first postoperative day follow-up visit, then she/he should be advised to do the following at home:

- Clean hands with soap and water. Carefully and gently remove the patch and then clean the operated eye starting from the nasal side in a horizontal stroke using clean towel and warm water, and soap if available. Tell the patients that the operated eyelid will appear swollen, but that this is normal.
- Use light pressure only.
- Dry with clean towel or cloth and then apply tetracycline eye ointment between the lower eyelid and the eyeball twice daily for two weeks.

**Figure 1:**
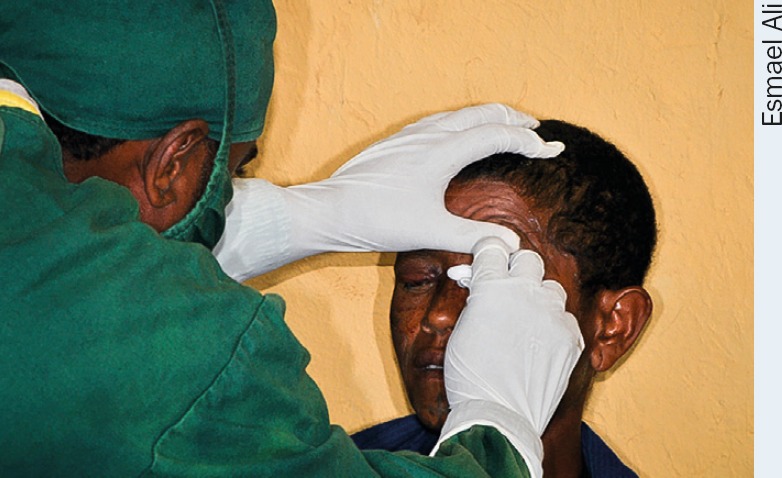
First day postoperative care

Patients may resume day-to-day activities on the second postoperative day. It is important to say this to the patients preoperatively as fear of not being able to work after surgery is among the main reasons deterring patients from accepting trichiasis surgery. However, they should be advised about the importance of keeping the wound clean and avoiding frequent contact with unwashed hands. Activities that may excessively expose the operated eyelid to dust should be avoided for few days. In some trachoma areas there is a belief that the smoke from a fire during cooking ‘would make the trichiatic lashes return’. Patients should be advised that they can resume cooking, however they should know that the fire smoke may create irritation and itchiness in the eye. They must avoid touching the eye and rubbing it as this may introduce infection as well as disrupt the surgical sutures and correction.

Patients should be advised to come back to the clinic if any of the following occur.

- Postoperative bleeding (visible through the patch or after it has been removed).
- Signs of infection such as sustained swelling, redness, pain, fever or an itching sensation and discharge on the operated lid.
- A sensation of stabbing pain or discomfort and excessive tearing. This is usually due to a suture fragment or a broken eyelash tucked into the tarsal conjunctiva during the time of surgery; this would constantly rub the cornea and/or conjunctiva during blinking.
- The presence of any lashes touching the cornea at any time after surgery.
- When the operated eyelid does not close properly or there is an eyelid contour abnormality or uncosmetic irregularity.
- Granuloma, a sessile fleshy growth on the tarsal conjunctiva that may occur between 1 and 6 months after surgery.

**Tropical Data: a new service for generating high quality epidemiological data**

Tropical Data – a WHO-led service helping national programmes collect and achieve more with their data-was launched on July 20, 2016.

Good quality prevalence data are essential for countries to plan, implement and monitor programmes attempting to eradicate, eliminate or control tropical diseases. The trachoma community has just completed the Global Trachoma Mapping Project, the largest infectious disease mapping effort ever completed, involving the examination of 2.6 million people. Now, the systems and methodologies used for the Global Trachoma Mapping Project have been further refined to create Tropical Data. Tropical Data supports countries to develop population-based survey protocols that are consistent with WHO recommendations, train and certify field teams using standardised training materials and certified trainers, create appropriate budgets, project-manage training and fieldwork, maximise the fidelity of data capture in the field through use of a purpose-built Android smart-phone-based app, secure data through encryption (both at rest and in transit) apply built-in data quality assurance and quality control processes, independently and consistently clean data (with logging of all changes and retention of original raw datasets alongside cleaned ones), automatically analyse cleaned data to generate key prevalence metrics, maintain country ownership and control of data (with constant password-protected access of health ministry-designated officials to their data from the moment that they are uploaded and health ministry review and approval steps hard-wired into the data pathway), and apply survey outputs. The net result is streamlined, cost-efficient generation of data of the highest quality that can be rapidly used for programmatic decision-making.

**Figure:**
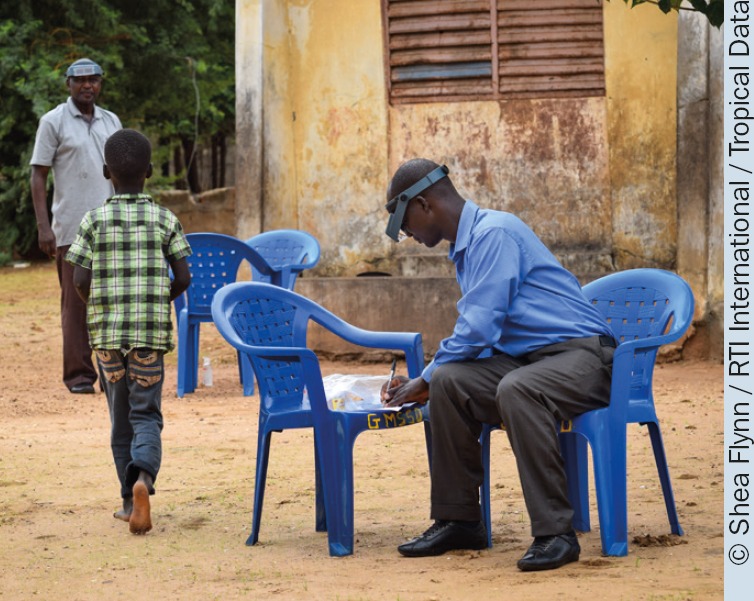
An inter-grader agreement test for Tropical Data Master Graders. SENEGAL

Tropical Data supports baseline, impact and surveillance surveys. It can be deployed quickly and scaled to local need. As the name suggests, Tropical Data intends to open up its service to other diseases in the coming months and years. The goal is to see the control, elimination or eradication of multiple diseases in our lifetime.

For more information, visit our website **www.tropicaldata.org**

**P J Hooper** (International Trachoma Initiative, Task Force for Global Health), **Tom Millar** (Sightsavers), **Lisa A Rotondo** (RTI International) and **Anthony W Solomon** (World Health Organization)

**Acknowledgements:** Tropical Data has been made possible thanks to assistance from the ENVISION project, led by RTI International and funded by the United States Agency for International Development; the International Trachoma Initiative; The Queen Elizabeth Diamond Jubilee Trust; Sightsavers; the United Kingdom's Department for International Development; and the World Health Organization.

